# A Comprehensive Review on the Role of Genetic Factors in Neuromyelitis Optica Spectrum Disorder

**DOI:** 10.3389/fimmu.2021.737673

**Published:** 2021-10-05

**Authors:** Soudeh Ghafouri-Fard, Tahereh Azimi, Mohammad Taheri

**Affiliations:** ^1^ Department of Medical Genetics, School of Medicine, Shahid Beheshti University of Medical Sciences, Tehran, Iran; ^2^ Men’s Health and Reproductive Health Research Center, Shahid Beheshti University of Medical Sciences, Tehran, Iran; ^3^ Skull Base Research Center, Loghman Hakin Hospital, Shahid Beheshti University of Medical Sciences, Tehran, Iran

**Keywords:** genetics, HLA, association, neuromyelitis optica spectrum disorder, expression

## Abstract

Neuromyelitis optica spectrum disorders (NMOSD) comprise a variety of disorders being described by optic neuritis and myelitis. This disorder is mostly observed in sporadic form, yet 3% of cases are familial NMO. Different series of familial NMO cases have been reported up to now, with some of them being associated with certain HLA haplotypes. Assessment of HLA allele and haplotypes has also revealed association between some alleles within HLA-DRB1 or other loci and sporadic NMO. More recently, genome-wide SNP arrays have shown some susceptibility loci for NMO. In the current manuscript, we review available information about the role of genetic factors in NMO.

## Introduction

Neuromyelitis optica spectrum disorders (NMOSD) comprise a variety of disorders being described by acute inflammatory responses in the optic nerve and spinal cord, i.e., optic neuritis and myelitis, respectively (1). NMO is mostly triggered by IgG autoantibodies against aquaporin 4 (AQP4) (2). AQP4 monomers comprise six transmembrane helical domains and two small helical parts around a thin aqueous pore (3). These monomers lump together to make corresponding tetramers with the ability of being aggregated in cell plasma membranes. The constructed supramolecular collections are named as orthogonal arrays of particles (OAPs) (3). AQP4 is the supreme ample water-channel protein in the central nervous system (CNS) (1). A number of NMO patients do not have AQP4-IgG, yet they have IgG antibodies against myelin oligodendrocyte glycoprotein, a glycoprotein in the outer myelin sheath of CNS neurons (4).

Following the discovery of AQP4-specific proliferative T cells in NMO patients, it has been recognized that AQP4-specific T cells exhibit Th17 features and display molecular mimicry with a peptide sequence encoded by the commensal bacterium *Clostridium perfringens*. Further studies have revealed distinct features of gut microbiota in NMO cases versus both multiple sclerosis (MS) cases and healthy subjects (5).

Although this disorder has some similarities with MS, it is important to distinguish between these two conditions, particularly at early stages of the disorder, since therapeutic modalities for these disorders are different (6). Most importantly, a number of prescribed agents for MS might be harmful for patients with NMO (7, 8). NMO and MS can be differentiated through assessment of NMO antibody. Although the existence of cerebral lesions has been formerly regarded as a criterion for differentiation between these two conditions, it is currently acknowledged that these lesions do not exclude NMO. In fact, with the advent of NMO antibody assessment techniques, some cases diagnosed as MS for a long time have been found to have NMO (9).

Typically, NMO manifests around the ages of 35 to 45 years, yet less than 20% of cases occur in children, and elderlies account for 18% of cases. NMO is recognized as a condition with female predominance. Although 70% to 90% of total NMO patients are female, such sex bias is not seen in children (6, 10). In NMO-AQP4 cases, gender influences both age at disease onset and site of attack (11).

NMO is most probably a complex multifactorial disorder. Most cases of this disorder are sporadic, yet 3% of cases are familial (12). A previous meta-analysis of whole-genome association studies in NMO has shown association of AQP4-IgG positive NMO with two independent signals in the MHC region. Notably, one of these signals has been suggested to be related with structural variations in the complement component 4 region. Moreover, a significant causal effect has been found between AQP4-IgG positive NMO and recognized risk variant for systemic lupus erythematosus (SLE). Most notably, such causal link has not been observed with MS risk variants (13). A number of other studies have reported an association between genetic variants and gene expressions alterations and NMO. In the current manuscript, we review available information about the role of genetic factors in NMO.

## Family Studies

Familial and sporadic NMO are similar in terms of clinical manifestations, age onset of disease, gender-based effects, and proportion of AQP4-IgG positive cases (12). A pioneer study in this field has reported occurrence of NMO in identical twin sisters at the ages of 24 and 26, respectively (14). A subsequent study reported NMO manifestations such as sudden loss of vision and transverse myelopathy in two sisters at the age of 3. Notably, HLA haplotyping revealed a shared haplotype between these two sisters, yet an unaffected sib also had this haplotype (15). More recently, a group of researchers described a series of familial NMO cases including siblings, parent–child, and aunt–niece pairs, more than 80% of them being female. A number of reported cases had either maternal or paternal transmission. More than 75% of cases had AQP4-IgG. About half of cases had clinical manifestations or serologic markers of another immune-related condition. The observed familial transmission of NMO suggested a complex genetic etiology for this disorder (12). A number of other studies also reported familial clustering of NMO cases, with some of them reported the presence of a shared haplotype among affected cases. **Table 1** summarizes the results of family studies in NMO.

**Table 1:** Summary of the results of family studies in neuromyelitis optica [HLA, human leukocyte antigen, AQP4-Ab, aquaporin-4 antibody (NMO-IgG)].

Cases	Population	Age at onset (years)	AQP4-Ab	HLA	Environmental factors	Year	Comments	Ref
Identical twin sisters	American	24 and 26	__	__	They had a history of bronchitis, measles and chickenpox.	1936	__	(14)
2 sisters	American	3 (similar)	__	HLA-A1, 2 BW35, W40, BW622 ————— HLA- A1, X BW35, YBW62 (Shared haplotype)	__	1982	Severity of the disease was different between cases. They had an unaffected sister until 3 years old, with a shared HLA haplotype.	(15)
2 sisters	Japanese	59 and 62	__	HLA-A 2/33, B 39/44, Cw7/2, DR 4/6, DQ 1/3 ————— HLA-A26/33, B 44/62, Cw3/2, DR 6/12, DQ 1/2, DP1/2, (Shared haplotype) HLA-DRB1*1202, 1302, DQB1*0604, 0301, DPB1*0501,0402	__	2000	One of the cases had rheumatoid arthritis since she was 30.	(16)
Mother and daughter	Unknown (published from USA)	62 and 29	Positive in mother (test was not performed in daughter)	__	__	2007	The daughter had a history of myasthenia gravis in childhood.	(17)
2 sisters, Niece–aunt, Daughter–mother, Daughter–father, Brother–sister, Monozygotic twin sisters, Son–mother	Lao, African American, Mexican, Brazilian, Vietnamese, Korean, African Caribbean	Different	76% of patients were NMO-IgG positive	__	__	2010	48% of cases had clinical or serologic sign of another autoimmune disorder (thyroid disease, T1DM, Sjögren syndrome, CIDP and psoriasis).	(12)
2 sisters	Japanese	25 and 26	Positive	HLA- A*31, B*61, *51, DRB1*0802, and DPB1*0501	The same until first episode of disease	2011	Genetic factors may influence age at onset of disease while environmental factors might be related to relapsed courses.	(18)
Mother and daughter	Unknown (published from USA)	78 and 38	positive	__	Mother had history of recurrent urinary tract infections	2015	There was genetic anticipation in familial NMO.	(19)
2 sisters	Unknown (report from USA)	3 and 3.5	positive	__	__	2016	NMO can have extended remission course but a persistent tendency to relapse.	(20)
Mother and daughter	Taiwanese	39 and 22	positive	HLA-DRB1*03 and HLA-DPB1*04	__	2019	__	(21)

## HLA Studies

An HLA genotyping study in seropositive Brazilian NMO patients has revealed some susceptibility loci for NMO, most importantly HLA-DRB1*04:05 and *16:02. A number of alleles within HLA class I showed association with NMO, yet this association did not remain significant after corrections for multiple comparisons (22). Another study in Afro-Caribbean NMO cases has shown higher frequency of HLA-DRB1*03 in NMO patients. On the other hand, HLA-DRB1*15, but not DRB1*03 allele has been recognized as a susceptibility locus for MS. In brief, distribution of HLA-DRB1 and DQB1 has been different among NMO and MS cases in this population (23). Another study in seropositive Brazilian NMO patients has shown overrepresentation of the HLA-DRB1*03 allele group in NMO cases compared with unaffected individuals. On the other hand, MS patients have shown higher frequency of the HLA-DRB1*15 allele group. DRB3 and DRB5 have had higher frequencies in NMO and MS cases, respectively (24). Another study has confirmed overrepresentation of HLA-DRB1*03 and HLA-DRB1*10 alleles in another group of Brazilian NMO patients compared with controls, in spite of no significant overrepresentation of MS-associated alleles (25). In addition, the DR3 and DR15 haplotypes have been found to be more common in NMO and MS, respectively. The association between HLA-DRB1*03:01 allele and NMO has not been dependent on seropositivity (26). In a study in Japanese patients, HLA-DRB1*08:02 and HLA-DRB1*16:02 have been found as risk loci, while HLA-DRB1*09:01 has been a protective allele (27). **Table 2** shows the results of HLA studies in NMO cases in different populations.

**Table 2:** HLA studies in neuromyelitis optica (SSP-PCR, sequence-specific primers–polymerase chain reaction; PCR-SSO, polymerase chain reaction–sequence specific oligoprobes; SBT, sequencing-based typing; MOG-Ab, myelin oligodendrocyte glycoprotein antibody).

HLA regions	Number of samples	Population	Source of sample/assay methods	Associations	Year	Ref
HLA-A, B, C HLA-DRB1, DQB1, DPB1	15 NMO patients and 606 healthy controls	Southern Brazilian	Peripheral blood/Sanger sequencing	There was significant association between HLA-DRB1*16:02, *04:05, C*15:02 alleles and NMO susceptibility.	2019	(22)
HLA-DRB1, DQB1	42 NMO patients and 150 healthy controls	French Afro-Caribbean	Peripheral blood/PCR-SSO	There was significant association between HLA-DRB1*03 alleles and NMO disease.	2010	(23)
HLA-DRB1, 3, 4 and 5	27 NMOSD patients and 28 healthy controls	Mulatto Brazilian (Ribeira˜o Preto)	Peripheral blood/PCR-SSP	HLA-DRB1*03 and DRB1*10 alleles were overrepresented in NMOSD patients compared to controls.	2009	(24)
HLA-DRB1	35 NMO patients and 99 healthy controls	Brazilian (Mexico City)	Peripheral blood/PCR-SSP	HLA-DRB1*03 and DRB1*10 alleles were more common in NMO cases compared to controls.	2016	(25)
HLA-DRB1, DQA1 and DQB1	65 NMO patients and 100 healthy controls	Brazilian (Rio de Janeiro)	Peripheral blood/PCR-SSO and SSP	HLA-DRB1*01:02, 03:01, DQB1*02:01 and DQA1*01:05 alleles were more common in NMO cases compared to controls. DRB1*03:01- DQA1*05:01/3/5-DQB1*02:01, DRB1*01:02-DQA1*01:01-DQB1*05:01 and DRB1*10:01-DQA1*01:04/5-DQB1*05:01 haplotypes were associated with NMO.	2017	(26)
HLA-A, B, C, DRB1 and DQB1	71 NMO patients and 97 healthy controls	Mexican	Peripheral blood/SBT	Risk HLA alleles for NMO: DQB1*03:01, DRB1*08:02, DRB1*16:02, DRB1*14:06, DQB1*04:02, B*35:14, B*39:06 and protective alleles include: DQB1*03:02, DQB1*02:02, DRB1*04:07, DRB1*07:01 and B*39:05	2020	(28)
HLA-A, B, DQA1, DQB1, DRB1, and DPB1	39 NMO, 6 patients at risk of NMO, and 100 healthy controls	French Caucasian	Peripheral blood/PCR-RFLP and PCR-SSP	HLA-DQA1*102, * 501, DQB1*0201 DRB1*03 alleles were significantly associated with NMO. There was no correlation between distribution of HLA alleles and IgG antibody subgroups	2009	(29)
HLA-DRB1	22 NMO patients and 225 healthy controls	Spanish Caucasian	Peripheral blood	HLA-DRB1*10 allele was significantly associated with NMO disease.	2011	(30)
HLA-A, B, C, DRA, DRB1, DQA1, DQB1, DPA1, DPB1, E, F, G, DOA, DOB, DMA, and DMB	31 NMOSD patients and 429 healthy controls	Japanese	Peripheral blood/NGS-based HLA genotyping	HLA-DQA1*05:03 allele had the most association with NMOSD.	2019	(31)
HLA-DRB1 and DPB1	77 NMO, 39 NMOSD patients and 367 healthy controls	Japanese	Peripheral blood/PCR-SSO	Higher occurrence of HLA-DRB1*1602, DPB1*0501 and lower occurrence of DRB1*0901 alleles were associated with anti-AQP4 antibody positive patients.	2012	(32)
HLA-DRB1 and DPB1	165 NMOSD patients	Japanese	Peripheral blood/SSO (Luminex)	HLA-DRB1*08:02 and DPB1*05:01 alleles were associated with disease and DRB1*09:01 was protective allele in NMOSD.	2021	(33)
HLA-DRB1 and DPB1	184 NMOSD patients and 317 healthy controls	Japanese	Peripheral blood/PCR- SSO	HLA-DRB1*08:02, -DRB1*16:02 alleles were associated to NMO whereas DRB1*09:01 allele was protective factor.	2020	(27)
HLA-DRB1 and DPB1	38 NMOSD AQP4-Ab^+^ patients and 125 healthy controls	Japanese	Peripheral blood/PCR-SSO	HLA-DPB1*0501 allele was associated with NMOSD and reinforced presence of anti AQP4-Ab	2008	(34)
HLA-DRB1	61 NMO and 32 NMOSD patients and 300 healthy controls	Indian	Peripheral blood/PCR-SSP	HLA-DRB1*03 allele was significantly associated with disease and persist associated with anti-AQP4 subtype. HLA-DRB1*10 allele was trended to associated with disease.	2015	(35)
HLA-DP	86 NMOSD patients and 29 healthy controls	Chinese	Peripheral blood/flow cytometry and real-time PCR	HLA-DPB1*0501 allele was associated with NMOSD through affect transcription levels of HLA-DP gene in antigen presenting cells.	2019	(36)
HLA-DQA1, DQB1 and DRB1	41 NMO patients and 200 healthy controls	Caucasian (Danish)	Peripheral blood/PCR-SSO	HLA-DQB1*0402 allele was significantly associated with NMO disease. There were no significant differences in HLA distributions between anti-AQP4 subtypes.	2011	(37)
HLA-DQ and DR	8 NMOSD patients with AQP4-Ab, 10 with MOG-Ab and 14 healthy controls	Swiss	Peripheral blood/PCR-SSP	HLA DQB1∗02, DRB1∗01 and DRB1∗03 alleles were significantly associated with AQP4-Ab^+^patients.	2020	(38)
HLA-A, B, C, DQA1, DQB1, DRB1 and DPB1	5 NMO patients	Southern Finnish	Peripheral blood/NGS and SSP	HLA-DPB1*0501 allele was associated with AQP4-Ab^+^ NMO patient.	2015	(39)
HLA-A, -B, -Cw, DRB1, DQB1 and DRB3/4/5	85 patients (include 43 MOG-IgG and 42 AQP4-IgG seropositive) and 5,604 healthy controls	Dutch	Peripheral blood/SSO (Luminex) and PCR-SSO	HLA-A*01, B*08, and -DRB1*03 alleles were significantly associated with AQP4-IgG NMOSD. There was no association of MOG-IgG cases with HLA alleles.	2020	(40)
HLA-DRB1 and DQB1	35 NMO patients and 74 healthy controls	Israeli Muslim	Peripheral blood/PCR-SSO, Luminex technology and PCR-SSP	There was a significant positive association of HLA-DRB1*04:04 and DRB1*10:01 alleles and negative association of HLA-DRB1*07 and DQB1*02:02 alleles with NMO.	2016	(41)
HLA-DRB1 and DPB1	30 NMO patients and 93 controls	Southern Han Chinese	Peripheral blood/SBT	The frequency of HLA-DRB1*1602 and DPB1*0501 alleles was significantly higher in NMO AQP4-Ab-positive patients. DRB1*0901 allele had lower frequency in disease.	2010	(42)

## Genomic Studies

Whole-exome sequencing (WES) has facilitated identification of risk loci for NMO. Application of this method in addition to HLA sequencing in seropositive NMO cases of Chinese origin has shown significant association between HLA-DQB1*05:02 and NMO. Additionally, the frequency of “HLA-DQB1*05:02-DRB1*15:01” haplotype has been higher in the NMO group compared with controls. Besides, this study has shown higher frequency of loss-of-function mutations in *NOP16* in these patients compared with healthy subjects. The G390R of IgG1, which decreases the threshold for BCR activation, has been another NMO-associated variant. Notably, most of the NMO-associated genetic factors have been enriched pathways related with nervous system and immune responses (43).

Another genome-wide study using an SNP array has identified the rs1964995 in the MHC region as a risk locus for NMO. Notably, three MS-associated variants have also been found to be associated with NMO. A variant within *KCNMA1* gene has been associated with disability score as well as presence of transverse myelitis (27).

The importance of copy number variations (CNVs) in conferring risk of NMO has been previously assessed using a genome-wide method. The majority of identified CNVs have been located at TCRγ and TCRα regions. These CNVs have been mostly deletions with sizes of 5 to 50 kb. Since they have been only in the peripheral blood T cells, it has been deduced that they are most probably somatically acquired CNVs. Moreover, it has been an association between the presence of CNVs in NMO cases and seronegativity for AQP4-IgG or low antibody titer (44).

Several SNPs within *AQP4* gene have been genotyped in NMO cases to find possible risk loci for this condition in different ethnic groups. For instance, Matiello et al. have compared genotype frequencies of 8 SNPs within *AQP4* gene in sporadic and familial NMO cases as well as healthy controls. One of these SNPs has been found to be associated with risk of NMO. Moreover, two missense mutations at Arg19 have been found in three NMO patients. The authors have reported that apart from one infrequent SNP, no other examined SNP or haplotype has been linked to NMO, possibly excluding the importance of *AQP4* variants in conferring risk of NMO (45). Qiu et al. have also genotyped eight SNPs in *AQP4* in a group of AQP4-IgG-positive NMO cases. They have shown associations between a number of SNPs and clinical manifestations of NMO such as extensive transverse myelitis, optic neuritis, or simultaneous systemic autoimmune disorders (46). **Table 3** shows the results of genomic studies in NMO cases.

**Table 3:** Genomic studies in neuromyelitis optica.

Genes	Number and type of samples	Population	Source of samples/assay method	Associations	Ref
Exome sequence	228 AQP4+ NMOSD patients and 1,400 healthy controls	Chinese	Peripheral blood/whole exome sequencing	The result represented most variants related to immune and nervous system. Significant variation in HLA region specifically DQB1, DQA2, and DQA1 was shown and the most significant allele was HL A-DQB1*05:02. NOP16 mutation and g G1-G390 R variant were also more common in patients.	(43)
Genome wide SNPs	203 NMO patients and 1782 healthy controls	Japanese	Peripheral blood/GWAS (HumanOmniExpress-12 BeadChip)	- 46 SNPs were identified around the *AQP4* gene - rs1964995 in the MHC region was the most associated SNP in NMO. - rs7186814 in chr 16 was associated SNP out of MHC region. - Three variants of MS risk were associated with NMO susceptibility. rs6677309 [*CD58*], rs1813375 [*EOMES – CMC1*], and rs694739 [*PRDX5 – CCDC88B*]) - rs1516512 in the *KCNMA1* was associated with EDSS and transverse myelitis.	(27)
Copy number variations	Identification phase: 135 NMO/NMOSD patients and 288 healthy controls Confirmation phase: 76 NMO/NMOSD patients and 790 healthy controls	Japanese	Peripheral blood/GWAS (high density SNP microarray) and qPCR	- 24 CNVs were significantly associated to NMO/NMOSD. They were mostly located on chr14. - A CNV deletion between 22,762,299 and 22,775,479 in TRA were prevalence in 13.27% of NMO. - Other CNVs were located on chr6 and 18. - Patients carrying CNVs tended to be AQP4-Ab^-.^	(44)
8 SNPs in *AQP4*	177 sporadic NMO patients, 14 familial NMO patients, and 1,363 matched healthy controls	African American, Latino, Asian, Arabic and unknown	Peripheral blood/TaqMan-based assay and sequencing	On of *AQP4* SNPs (NC 18.8; chromosome pos. 22695167: T>A) was associated with disease. Two different allelic missense mutations, Arg19 (R19I and R19T) was specific to NMO.	(45)
8 SNPs in *AQP4*	208 NMO patients (AQP4-Ab^+^) and 204 healthy controls	Chinese	Peripheral blood	- rs1058424 (A/T) and rs3763043 (C/T) were correlated with LETM. - rs1058424 (A/T), rs335929(A/C), and rs151244(C/T) were correlated with optic neuritis. - rs6508459 and rs3763040 were associated with concurrent systemic autoimmune diseases.	(46)
6 SNPs in *AQP4*	62 NMOSD patients and 109 healthy controls	Northern Han Chinese	Peripheral blood/high-resolution melting	There were no substantial differences in frequency of alleles between NMO/NMOSD and controls.	(47)
*AQP4* exon 1,2,3,4,5	72 NMO patients	Chinese	Peripheral blood/sequencing	- 6 SNP sites in exons 2 and 5 were identified in NMO patients. - AQP4-Ab serum levels were significantly different between R108T/I110N, E280R/D281R, E317M variants and original cell line.	(48)
*AQP4* sequence and 10 SNPs	64 NMO and 58 NMOSD for sequencing 111 NMO, 97 NMOSD and 204healthy controls for genotyping	Chinese	Peripheral blood/sequencing and PCR-LDR	A/T genotype of rs1058424 and C/T genotype of rs3763043 were more frequent in NMO.	(49)
*AQP4* exon 1,2,3,4,5	27 NMO patients and 40 healthy controls	Han Chinese	Peripheral blood/sequencing	rs72557968 in exon 2 was identified in one NMO-IgG^+^ patient. The mutated sequence correlated with higher AQP4-Ab expression.	(50)
*AQP4* promoters	18 NMO patients and 39 healthy controls	Southern Han Chinese	Peripheral blood/PCR and sequencing	- Polymorphism at −1003 bp (A-G) position of promoter 0 was associated with AQP4-Ab presence. - Polymorphisms between −401 bp and−400 bp locations of promoter 1 were more frequent in NMO compared to controls.	(51)
*AQP4* exons and 5 SNPs	16 AQP4-Ab^+^ NMO patients and 255 healthy controls	Japanese	Peripheral blood/sequencing and TaqMan assay	T allele of rs2075575 in promoter region was significantly more frequent in NMO and led to downregulation of *AQP4* gene.	(52)
35 non-MHC MS risk loci	110 NMO patients and 332 healthy controls	Southeastern China	Peripheral blood/MALDI-TOF MS	Only rs1800693 in the *TNFRSF1A* locus tended to be associated with NMO.	(53)
Thiopurine nucleotides and SNPs in *MTHFR* *TPMP, SLC29A1*, *SLC28A1*, *ABCB1*, *SLC28A3*, *HLA*, *ABCC4*, *SLC28A2*	32 NMO patients	Chinese	Peripheral blood/LC-MS/MS, MassARRAY and multiple SNaPshot techniques	In *SLC28A3* gene, rs10868138 and rs12378361 were correlated with higher and lower erythrocyte concentration of 6-TGNs, respectively. rs507964 in *SLC29A1* was associated with lower erythrocyte concentration of 6-MMPNs and 6-MMPNs:6-TGNs ratio.	(54)
*CYP27B1*: rs12368653 rs10876994 rs118204009 rs703842 *CYP24A1*: rs2248359	110 NMO patients and 294 healthy controls	Han Chinese	Peripheral blood/MassARRAY system and sanger sequencing	rs703842 and rs10876994 were significantly associated with NMO compared to controls.	(55)
11 SNPs in *CYP7A1*	90 NMO patients and 240 controls	Korean	Peripheral blood/Bead Express	- rs3808607 and rs1457043 were associated with NMO. -”G/G” genotype of rs3808607 had a higher protective effect on the risk of disease.	(56)
Promoter region of *CYP7A1*	89 NMO patients and 325 controls	Han Chinese	Peripheral blood/sanger sequencing	−204A>C (rs3808607), −469T>C (rs3824260) and −208G>C were significantly associated with NMO.	(57)
*CD226*: rs763361	89 NMO patients and 129 healthy controls	Southern Han Chinese	Peripheral blood/sequencing	TT genotype of rs763361/Gly307Ser was associated with NMO susceptibility.	(58)
*CD58*: rs17426456 rs2300747 rs1335532 rs12044852 rs1016140 rs12025416	98 NMO patients (AQP4-Ab^+^) and 238 healthy controls	Korean	Peripheral blood/TaqMan assay	- 4 SNPs (rs2300747, rs1335532, rs12044852, and rs1016140) and 2 haplotypes in the *CD58* gene were significantly associated with NMO. - rs1016140 led to T-cell hyperactivity that caused AQP4-Ab access to CNS.	(59)
9 SNPs in *CD58*: rs1335532 rs10802189 rs56302466 rs472291 rs3789716 rs1335531 rs1335532 rs2300747 rs1016140	230 NMOSD patients and 487 healthy controls	Han Chinese	Peripheral blood/SNPscan Kit and PCR-LDR	- rs2300747, rs1335532, rs56302466, rs1016140, and rs12044852 were associated with NMOSD. - TAGCCCAA haplotype increased and TATTACGG haplotype reduced NMOSD risk.	(60)
21 SNPs in *CD6*, *TNFRSF1A* and *IRF8*	99 NMO patients and 237 healthy controls	Korean	Peripheral blood/TaqMan assay	rs12288280 in *CD6* gene and rs767455, rs4149577, rs1800693, and ht2, ht3 haplotypes in *TNFRSF1A* were significantly associated with NMO.	(61)
6 SNPs in *FCRL3*	150 NMO patients and 300 healthy controls	Chinese	Peripheral blood/MALDI-TOF-MS	G allele of -1901A>G and T allele of -658C>T polymorphism were significantly more frequent in patients	(62)
7 SNPs in *FCRL3*: rs7528684 rs11264799 rs945635 rs3761959 rs2210913 rs2282284 rs2282283	132 NMO patients and 264 healthy controls	Chinese	Peripheral blood/TaqMan assay and sequencing	Both allelic and homozygote model of s7528684, rs945635, rs3761959, and rs2282284 were significantly associated with NMO susceptibility.	(63)
9 SNPs in *GPC5*	99 NMO patients and 237 healthy controls	Korean	Peripheral blood/TaqMan assay	rs1411751, rs9523762 and BL1_ht3 haplotype of *GPC5* were significantly associated with NMO.	(64)
*MIF−173* rs755622	70 NMO patients and 60 healthy controls	Caucasian	Peripheral blood/PCR-RFLP	CC/GC genotypes in polymorphism were correlated with higher EDSS. These genotypes were more frequent in patients with both optic neuritis and myelitis. *MIF-173* in more associated with severity rather than susceptibility.	(65)
5 SNPs in *ATG5*: rs2245214 rs548234 rs573775 rs6568431 rs6937876	109 NMO patients and 288 healthy controls	Southern Han Chinese	Peripheral blood/MALDI-TOF-MS	CC genotype of rs548234 associated with NMO susceptibility while T allele of rs548234 and A allele of rs6937876 played a protective role in AQP4-Ab^+^ patients.	(66)
*PD-1.3* and *PTPN22* (1858 C/T)	41 NMO patients and 200 healthy controls	Danish Caucasian	Peripheral blood/sequencing and PCR-RFLP	-PD-1.3 A allele was associated with NMO. -There was no association between PTPN22 polymorphism and NMO.	(37)
*IL2RA*: rs2104286 rs12722489 rs7090512	75 NMO/NMOSD and 238 healthy controls	Japanese	Peripheral blood/TaqMan assay	There was no significant association between *IL2RA* polymorphisms and NMO.	(67)
*IL2RA*: rs2104286 rs12722489 *IL7RA*: rs6897932	67 NMO patients and 133 healthy controls	Southern Han Chinese	Peripheral blood/sequencing-based typing	G allele frequency of rs2104286 in *IL2RA* gene was significantly higher in NMO patients.	(68)
*IL-7:* rs1520333 rs1545298 rs4739140 rs6993386 rs7816065 rs2887502 *IL-7RA*: rs6897932	167 NMO patients (57 AQP4_Ab^+^) and 479 healthy controls	Southeastern Han Chinese	Peripheral blood/MassARRAY system and Sanger sequencing	rs6897932 in *IL-7RA* was significantly associated with NMO especially in AQP4-Ab^+^ patients.	(69)
13 SNPs in *IL7RA*	98 NMO patients and 238 healthy controls	Korean	Peripheral blood/TaqMan assay	There was no significant association with NMO.	(70)
*IL-17A*: rs2275913 *IL-17F*: rs763780	52 AQP4-Ab^+^ NMO patients and 131 healthy controls	Southern Han Chinese	Peripheral blood/sequencing	T allele of rs763780 was significantly more frequent in NMO patients compared to controls.	(71)
4 SNPs in *IRF5*	111 NMO patients and 300 healthy controls	Southeastern Han Chinese	Peripheral blood/MALDI-TOF-MS	There was no association between *IRF5* polymorphisms and NMO.	(72)
*CH25H*	14 NMO patients and 882 healthy controls	European and Asian	Peripheral blood/exome sequencing	c.51G>C, p.Q17H variant was identified in 2 Asian female patients.	(73)

## Expression Studies

Expressions of several immune-related genes have been assessed in NMO cases at transcript or protein levels. Moreover, a number of high-throughput sequencing strategies have been employed to assess expression of different subtypes of transcripts. For instance, lncRNA and mRNA profile has been assessed in these patients using microarray technique. Such type of analysis has led to the identification of more than 1,300 lncRNAs with differential expression between NMO cases and normal controls. Moreover, more than 700 mRNAs have been found to be differentially expressed between NMO cases and normal subjects. These genes have been functionally correlated with IL-23-related cascades, IFN-γ signaling, natural killer-κB pathway, and a number of other immune-related mechanisms (74). Another RNA expression profiling experiment has shown possible contribution of T-cell-related genes and the TNF/NF-kB cascade in the pathogenesis of NMO. Notably, IL7Ra (CD127) has been found to be downregulated in the circulation of NMO patients compared with control subjects. Moreover, transcription factors located in the upstream of CD127 and survival pathways in its downstream have been considerably downregulated. These expression changes have been accompanied by decrease in the quantities of naïve T cells, reduction of BID-mediated T-cell survival signaling and activation of cell apoptosis. Taken together, these observations indicate the importance of IL7Ra signaling in the pathoetiology of NMO (75). A high-throughput expression profiling in brain tissue samples obtained from an NMO patient as well as patients with Parkinson’s disease and amyotrophic lateral sclerosis has shown upregulation of more than 200 genes in brain lesions of NMO patients with the mostly upregulated ones being associated with immune response. Upregulation of IFI30, CD163, and SPP1 has also been confirmed by further RNA and protein-based techniques. Genes with high expression in NMO brain lesions has been functionally related with NF-κB and Blimp-1, indicating the importance macrophage-mediated inflammatory responses in the pathoetiology of NMO brain lesions (76).

With the aim of finding effective markers for the assessment of response of NMO patients to therapeutic options, Vaknin-Dembinsky et al. have assessed miRNAs profile in the blood of NMO patients before and following treatment with rituximab. They have reported upregulation of 14 miRNAs and downregulation of 32 miRNAs in NMO patients after treatment with rituximab. Moreover, they have shown higher levels of 17 miRNAs and lower levels of 25 miRNAs in untreated cases compared with healthy controls. Notably, rituximab could normalize expression of a number of these miRNAs, among them have been brain-specific or brain-enriched miRNAs. Cumulatively, circulatory miRNA profile can be used as a biomarker for therapeutic response (77).

The pleiotropic cytokine IL-6 is also implicated in the pathogenesis of NMO through enhancement of survival of plasmablasts, induction of release of antibodies against AQP4, disruption of integrity of blood–brain barrier and its functionality, as well as increasing differentiation and activity of proinflammatory T cells (78). Expression of this cytokine has been reported to be elevated in CSF and blood samples of NMO patients (79). **Table 4** shows the results of expression studies in NMO.

**Table 4:** Expression studies in neuromyelitis optica (NPSLE, neuropsychiatric systemic lupus erythematosus; ONND, other non-inflammatory neurological disorders; OND, other neurological disorders).

Genes	Number and type of samples	Population	Source of samples/assay method	Associations	Ref
lncRNA and mRNA profiles	16 NMO patients and 16 healthy controls	Chinese	Peripheral blood/microarray and qRT-PCR	Results represented differential expression of 1310 lncRNAs and 743 mRNAs in NMO compared to the healthy group, which is related to IL23-mediated signaling events, IFN-g signaling, NF-κB signaling pathway, chemokine receptors, GPCR ligand binding, and metabolic disorders of biological oxidation enzyme pathways.	(74)
526 immune-related genes	65 NMO patients and 37 healthy controls	Israelis	Peripheral blood/Nano String n Counter technology, RT-PCR, ELISA and Flow cytometry	Two main clusters were differentially expressed in NMO, namely, T-cell associated genes and NF-KB signaling genes. *IL-7Ra* was the most differentiated gene in the T-cell cluster that downregulated in patients. Furthermore, sIL7Ra and mIL7Ra isoforms were also lower in NMO especially AQP4+ samples.	(75)
mRNAs profile	1 NMO patient,1 Parkinson patient and 1 ALS patient	__	Post mortem Brain tissues/microarray, Real-time PCR, northern blot and Western blot	200 genes were significantly upregulated in NMO brain tissue which mostly related to immune regulation involved NF-kB and Blimp-1.	(76)
microRNAs profile	9 rituximab-responsive NMO patients,16 nontreated AQP4+ NMO patients and 15 healthy controls	Israelis	Peripheral blood/RNA-seq and real-time PCR	miRNA expression signatures were different in patients compared to healthy controls, also between rituximab responders and non-responders (e.g., miR-125). Rituximab changed the expression patterns similar to healthy controls (miR-7 and miR-124).	(77)
*QKI-V5* *QKI-V6* *QKI-V7*	23 NMO patients and 8 healthy controls	Israelis	Peripheral blood/qPCR and Western Blot	*QKI-V5* was significantly downregulated in patients.	(80)
MOG and AQP4 antibodies	215 NMOSD patients (adult and pediatric patients)	Japanese and Brazilian	Serum/cell-based assay (CBA)	64.7% of patients were AQP4-ab positive and 7.4% were MOG-ab positive. No one had both antibodies. MOG-ab+ patients had better prognosis.	(81)
AQP4-Ab25(OH) D_3_	29 NMOSD patients	Iranian	Serum/chemiluminescence immunoassay (LIAISON^®^) and immunofluorescence	25(OH) D3 serum levels were significantly lower in AQP4-Ab+ patients than patients with negative AQP4-Ab.	(82)
25(OH)D_3_	51 AQP4-ab positive NMOSD patients and 204 healthy controls	Korean	Peripheral blood/LC-MS/MS	25(OH)D_3_ levels were significantly lower in NMOSD patients compared to controls and its levels negatively correlated with EDSS scores.	(83)
25(OH) D_3_	19 NMO patients and 33 healthy controls	Indonesian	Serum/chemiluminescence immunoassay	There were no significant differences in 25(OH) D3 serum levels between NMO patients and healthy controls, and its levels were lower in patients who received corticosteroid treatments.	(84)
25(OH) D_3_	76 NMO/NMOSD patients and 54 patients with demyelination events	Thais	Peripheral blood/Elecsys^®^	There was no significant difference in 25(OH) D_3_ levels among patients with demyelinating disease	(85)
ANA Anti-dsDNA, anti-nucleosome, AQP4 and MOG antibodies Cytokines and chemokines	6 NMO patients with SLE diagnosis history (during relapse and remission) and 11 healthy controls	Hungarian	Serum/flowcytometry, ELISA and MSD Human V-Plex kit	AQP4-IgG1 was presented years before NMO diagnosis in SLE patients and correlated with the concentration of IFN-γ, CXCL10/IP-10, and CCL17/TARC. AQP4-IgG1, ANA, anti-dsDNA, and anti-nucleosome antibodies were increased during relapse. Autoantibody responses in NMO/SLE followed by Th1 responses.	(86)
27 cytokines/chemokines/growth factors	22 AQP4+ NMO patients and 32 NPSLE patients as a control group	Japanese	CSF/multiplex cytokine bead- based assay	IL-17, IL-2, FGF-basic, IL-5, IL-15, IL-9, IFN-gamma, IL-12, IL-10, IL-7, IL-13, TNF-a, and EOTAXIN levels were significantly lower in NMO compared to NPSLE.	(87)
27 cytokines/chemokines and growth factors	20 NMO/NMOSD patients and 18 OND patients as a control group	Japanese	CSF/Multiplexed fluorescent bead-based immunoassay	Upregulation in a group of Th17- and Th1-related proinflammatory cytokines/chemokines was represented in NMO. IL-6 and CXCL8 levels were significantly correlated with CSF protein concentration, cell count, neutrophil count, and EDSS.	(88)
27 cytokines/chemokines Th17 cell-associated cytokines	31 NMO patients and 18 ONND patients as a control group	Japanese	CSF and serum/	The CSF levels of IL-1 receptor antagonist, IL-6, IL-8, IL-13, IL-10, g-csf, and IP-10 were significantly higher in NMO, while only IL-6 level in serum has upregulation. CSF IL-6 level correlated with CSF cells and glial fibrillary acidic protein.	(79)
Th1, Th2, and Th17 cytokines	34 NMO patients (20 with IFN treatment) and 30 healthy controls	Taiwanese	Serum/cytometric bead array (CBA)	IL-2, IL-4, IL-6, IL-10, TNF-a, and IFN-g levels were significantly higher in patients. Patients who received IFN-g treatment had higher EDSS and IL-17 and lower IL-2 level.	(89)
Soluble CD27	31 NMO patients and 22 controls with noninflammatory neurological diseases	Chinese	CSF/ELISA	CD27 concentration was higher in NMO patients, especially in AQP4-IgG positive cases compared to the control group. Its higher level correlated with CSF total protein and worse disease disability.	(90)
Soluble Syndecan-1 (sSDC-1)	23 NMO patients and 16 healthy controls	Chinese	CSF and serum/ELISA	sSDC-1 concentration was higher in NMO patients. It had a positive correlation with disease severity and CSF levels of IL-6, IL-8, and IL-17.	(91)
B-cell subsets and T-cell subsets	22 AQP4+ NMOSD patients and 13 healthy controls	South Korean	PBMC/flow cytometry	Breg cells as IL-10-producing B (B10) cells were elevated in patients and correlated with AQP4-Ab.in addition, IL-17+Treg cells were higher in remission phase of disease.	(92)
IL-4	45 NMO patients and 45 healthy controls	Iranian	Serum/ELISA	IL-4 serum levels were increased in patients compared to healthy controls. Furthermore, gender (female) and AQP4-Ab were associated with IL-4 levels.	(93)
IL-4 IFN-gamma	28 NMO patients and 28 healthy controls	Afro-Brazilians	Plasma/ELISA	IL-4 higher levels in NMO represented of its crucial role in Th2 regulatory cell activation.	(94)
IL-2 IL-4 IL-6 IL-10 TNF-a IFN-c	17 NMO patients at relapse time and 21 OND patients	Japanese	CSF/FACS	Significantly higher levels of IL-6 identified in NMO patients.	(95)
IL-6	23 NMO patients and 19 healthy controls	Turkish	Serum and CSF/ELISA	Higher level of IL-6 was identified in sera and SCF samples of patients, particularly in seropositive AQP4-ab than negative type. CSF IL-6 level also correlated with disease severity and AQP4-ab levels.	(96)
IL-6	95 NMO patients (59 acute and 36 chronic phase) and 333 OND	Japanese	SCF/CLEIA	NMO patients had higher IL-6 levels of CSF. IL-6 represented high sensitivity and specificity for NMO diagnosis. Its concentration correlated with spinal cord lesion length and AQP4-Ab.	(97)
IL-6 sIL-6R	22 NMO patients and 14 healthy controls	Chinese	CSF/ELISA	IL-6 and sIL-6R levels were significantly higher in NMO. sIL-6R level also correlated with EDSS.	(98)
IL-6 GFAP	13 NMO patients and 20 ONND and 24 idiopathic CNS inflammatory patients as a control group	Japanese	CSF/CLEIA	CSF concentration of IL-6 and GFAP was significantly higher during initial NMOSD attacks. They could diagnosis early stage of NMO with high sensitivity.	(99)
IL-6 IL-1B	9 definite NMO patients and 8 limited forms of NMO with myelitis	Japanese	SCF/ELISA	Higher levels of IL-6 and IL-1B were shown in definite NMO patients compared to limited form.	(100)
IL-6 IL-5 IL-12 MOG-Ab eosinophil cationic protein (ECP)	8 NMO and 16 healthy controls	Argentines	SCF/ELISA and radioimmunoassay	Higher levels of IL-5, IL-6, MOG-ab, and eosinophil-related factors were identified in NMO patients.	(101)
IL-6 IL-17A Inulin sensitivity	56 NMOSD patients and 100 healthy controls	Iranian	Serum/ELISA	IL-6 and IL-17A serum levels were higher in patients. There was significant association between lower insulin sensitivity and higher level of IL-6.	(102)
HMGB1 TNF-α IFN-γ IL-17	29 NMO patients and 20 MS patients	Taiwanese	Plasma/ELISA	All parameters were significantly higher in NMO patients. HMGB1 level correlated with TNF-α, IFN-γ, and IL-17 levels. HMGB1 could diagnose and differentiate NMO with high sensitivity and specificity.	(103)
IL-6 IL-17 HMGB1	22 NMO patients and 14 healthy controls	Chinese	SCF/ELISA	HMGB1 was higher in CSF of NMO patients and correlated with IL-6 and IL-17 levels.	(104)
IL-6 HMGB1 GFAP	42 NMOSD patients and 30 ONND patients	Japanese	CSF and serum/ELISA and CLEIA	HMGB1 CSF levels were significantly elevated in NMOSD. its concentration correlated with other CSF parameters such as:IL-6 level, cell counts, protein levels, glial fibrillary acidic protein levels, and CSF/serum albumin ratio.	(105)
IL-6 IL-17A	31 NMO patients and 39 healthy controls	Iranian	Serum/ELISA	IL-6 serum level was lower than controls whereas IL-17 level was higher in NMO patients.	(106)
IL-6 IL-10 IL-17 IL-21	20 NMO patients and 20 healthy controls	Brazilian	PBMC/flow cytometry and ELISA	IL‐6, IL‐17, and IL‐21 were highly secreted from CD4+ T cells in patients. Disability scale in patients correlated with IL-6 and IL-21 levels. Furthermore, anti‐IL‐6R had potential to decreased Th17 cytokines.	(107)
IL-32α IL-6 IL-17A	26 NMO patients and 22 healthy controls	Chinese	Serum/ELISA	IL-32α serum level was higher in patients and correlated with EDSS, IL-6, and IL-17A levels.	(108)
IL-21, IL-6, IL-17, IL-10 TNF-α AQP4-antibody follicular helper T (Tfh) cells	35 NMO patients and 20 healthy controls		PBMC/flow cytometry and ELISA	IL-21, IL-6, and IL-17 concentrations were significantly higher in NMO while IL-10 was lower in patients. Tfh cells were higher in relapsing course and correlated with disease activity. Tfh cells were decreased under Methylprednisolone treatment.	(109)
Th17 CD8(+) T cells IL-17, IL-6, IL-21, IL-23 and TGF-β	14 NMO patients and 16 healthy controls		Peripheral blood/Flow cytometry and ELISA	Th17 cells and IL-17-secreting CD8(+) T cells were significantly higher in NM. Serum IL-17, IL-21 and IL-23 were significantly higher in NMO samples.	(110)
peripheral memory Th17 IL-17A IL-23	16 NMO patients and 16 healthy controls	Chinese	Peripheral blood/flow cytometry and ELISA	All the parameters were significantly higher in NMO and correlated with disease duration and relapse. Furthermore, intravenous methylprednisolone therapy could decrease IL-23 levels in patients.	(111)
IL-21	21 NMO patients and 16 healthy controls	Chinese	CSF/ELISA	CSF IL-21 level was significantly higher in NMO and correlated with humoral immune activity.	(112)
Th22 Th17 CD4+IL-22+IL-17A+T cells IL-22, IL-6, IL-21, IL-27 and IFN-γ	21 NMO patients and 12 healthy controls	Chinese	Peripheral blood/flow cytometry and ELISA	Proportions of Th22 and Th17 were significantly higher in patients.IL-21, IL-22, and FN-γ concentration were increased in NMO.	(113)
IL-4, IL-10, IL,9, IL-12, IFN-γ, IL-17, IL-23, and TGF-β	18 relapsing NMO (11 AQP4+ and 7 AQP4-) and 30 healthy controls	Turkish	Serum/ELISA	Th1-/Th17 responses were deregulated in patients. Serum IL-9 levels were higher in AQP4+ patients compared to negative serotype.	(114)
IL-37	31 NMO patients and 49 healthy controls	Iranian	Plasma/ELISA	IL-37 levels were significantly increased in patients and correlated with EDSS and disease duration.	(115)
IL-1β TNF-α NF-κB Bcl-2 PI3K/Akt MAP3K7 in CD4+ T cells	30 NMO patients and 25 healthy controls	Chinese	Peripheral blood/cytokine multiplex assay	NF-κB. Bcl-2 and MAP3K7 gene expression was upregulated in NMO. IL-1β and TNF-α levels were elevated and led to MAP3K7 induction, which promoted NF-κB expression related to survival of CD4+ T cells.	(116)
*IL-1β* *TNF-α* in CD14+ and CD16++ subset cells	15 NMO patients and 9 OND and 15 healthy individuals as controls	Chinese	Peripheral blood, CSF/Flow cytometry, qRT-PCR, ELISA	Specific subsets were increased in NMO patients along with total monocytes and they could be decreased *via* glucocorticoids therapy. In addition, *IL-1β* and *TNF-α* expression levels were significantly upregulated in NMO.	(117)
IL-1β TNF-α ENA 78	25 NMO patients and 20 healthy controls	Chinese	Plasma/MILLIPLEX^®^ map	IL-1β, TNF-α, and ENA 78 plasma levels were significantly increased in NMO. There was significant correlation between ENA 78 expression and EDSS in patients.	(118)
IL-21 and AQP4-Ab in memory T follicular helper (Tfh) cells	25 NMO/NMOSD patients (before and after treatment) and 17 healthy controls	Chinese	Peripheral blood and CSF/flow cytometry and ELISA	Tfh cell percentage and IL-21 were significantly increased in patients. Some subsets were correlated with AQP4-ab and WBC count in CSF. Corticosteroid therapy suppressed subtypes and IL-21 levels.	(119)
Cytokine and chemokine induced by specific AQP4 peptides/epitopes	14 NMO patients and 7 controls	Israelis	PBMC/cytometric bead array and flow cytometry	4 epitopes of AQP4 were showed in NMO and their specificity changed during disease course cell responses to these epitopes represented more IL-17 and IL-10 secretions.	(120)
BAFF-R CXCR5 VLA-4 B cell produce IL-10, IFN-γ circulating memory and regulatory cells	51 NMO patients and 37 healthy controls	Chinese	CSF/flow cytometry and ELISA	Proportions of CD19(+) CD24(high)CD38(high) regulatory B cell and producing IL-10 were significantly decreased in NMO, while BAFF and CXCL13 levels were higher in them. Furthermore, these proportions were lower in AQP-4 positive samples.	(121)
MMP9 TIMP1 TNF-*α* IFN-*γ* IL-10 oxidative stress markers	11relapsing NMO patients and 11 healthy controls	Cuban	Serum/ELISA and spectrophotometric methods	Downregulation of IL-10 and TNF-*α* and upregulation of oxidative stress markers were shown in the study.	(122)
MMP9 TIMP1 IL-17 IL-8 IP-10 MCP-1	13 NMO patients and 14 healthy controls	Japanese	Serum and CSF/ELISA	Serum MMP9 level was significantly higher in NMO and its concentration correlated with CSF IL-8, CSF/serum albumin ratio and EDSS. MMP9 played a crucial role in BBB disruption.	(123)
9 MMPs 4 TIMPs 14 cytokines	29 NMO patients and 27 OND patients	Japanese	Serum, CSF and post-mortem brain tissue/multiplex assay and immunohistochemistry	MMP-2, TIMP-1, IL-6 levels, and MMP-2/TIMP-2 ratio in CSF were significantly increased in NMO.MMP-2 concentrations correlated with IL-6 levels and BBB permeability.	(124)
MMP2 MMP9	14 seropositive AQP4 NMOSD patients and 10 healthy controls		Serum/ELISA	There were no significant differences in MMP2 and MMP9 levels in NMOSD compared to controls.	(125)
AQP4-Ab TNF-α GFAP CXCL12	40 NMOSD patients (20 good and 20 poor recovery)	Chinese	CSF and serum/immunofluorescence and ELISA	Patients with poor recovery had higher AQP4-Ab serum level. Furthermore, AQP4-Ab in good recovery patients was even lower than poor group after treatment. CXCL12 level was significantly lower in poor recovery group and negatively correlated with AQP4-Ab level. It was also related to TNFα and GFAP CSF levels.	(126)
Anti-AQP4 Anti-AQP1 Anti-MOG	18 NMOSD and 8 healthy controls	Spanish	Serum/Immunofluorescence Assay and ELISA	According to the results, only anti-AQP4 antibodies could act as a biomarker in NMOSD diagnosis, and its level was not correlated with disease progression.	(127)
Anti-AQP4	16 NMO patients and 30 healthy controls	Italian	Serum/Western blot	Western blot assay could distinguish immunoreactivity of AQP4 isoforms.	(128)
*OX40* (CD134)	20 NMO patients and 20 healthy controls	Iranian	Peripheral blood/RT-PCR and ELISA	OX40 expression level was downregulated in patients compared to controls, while there were no significant differences in serum levels.	(129)
G6PD	50 NMO patients and 65 healthy controls	Iranian	Serum/ELISA	G6PD serum level was significantly lower in NMO patients compared to controls.	(130)
AQP4 isoforms	1 NMO patient and 12 not neurologic patients as control group	__	Post mortem CNS tissue/sequencing and Real time-PCR	AQP4 isoforms expression pattern correlated with NMO disease localization and the highest mRNA M1:M23 ratio was identified in optic nerve and spinal cord.	(131)

## *In Vitro* Studies

A number of *in vitro* studies have appraised the functional mechanisms of development of NMO. In an effort to find the impact humoral factors on astrocyte injury in NMO, Haruki et al. have conducted a series of experiments on immortalized human primary astrocytes. Moreover, they assessed the effect of TY09 human brain microvascular endothelial on the quantity and localization of AQP4 protein in astrocytes. Serum samples of NMO patients have been shown to induce cytotoxic effects on AQP4-expressing astrocytes. Moreover, these serum samples could decrease AQP4 expression at both mRNA and protein levels, while increasing release of TNF-α and IL-6 from astrocytes. Experiments in an *in vitro* BBB model has shown localization of AQP4 protein at the astrocytic membrane following co-culture with TY09, in contact with these cells (132).

Sera samples of these patients or even NMO-IgG have also been shown to rapidly downregulate AQP4 levels on the surface of astrocytes. Astrocytes treated with NMO-IgG, IL-6/R, and NMO-IgG + IL-6/R have shown over-production of IL-6 transcripts. Moreover, NMO-IgG could elicit alterations in gene transcription *via* the JAK/STAT3 pathway. Cumulatively, NMO-IgG has been reported to induce the JAK1/2/STAT3 pathway in astrocytes, representing a crucial event in the pathoetiology of NMO. Besides, suppression of JAK1/2 signaling might be a therapeutic modality for NMOSD (133).

Another *in vitro* study has shown similar magnitude of lymphoproliferation and cytokine profiles in peripheral blood mononuclear cells of NMO cases and healthy controls in reponse to *Staphylococcus aureus* and *Candida albicans*. However, NMO-originated *Escherichia coli*-induced cell cultures have exhibited higher proliferation of CD4+ T cells in association with higher production of IL-1β, IL-6, and IL-17. IL-10 release has been lower in NMO-derived cells compared with controls. Notably, the *in vitro E. coli*-stimulated expressions of IL-6 and IL-17 have been correlated with neurological debilities. Overproduction of Th17-associated cytokines has been associated with the production of IL-23 and IL-6 by LPS-stimulated monocytes. Consistently, LPS levels have been higher in the plasma samples of NMO cases. Therefore, increase in Th17 type response to *E. coli* might contribute in the pathogenesis of NMO (134). **Table 5** shows the results of *in vitro* mechanistical studies in NMO.

**Table 5:** *In vitro* studies (BMECs, brain microvascular endothelial cells).

Genes and cells	Number and type of samples	Population	Source of samples/assay method	Results	Ref
AQP4IL-6TNF-aCytotoxicity	5 AQP4+ NMO patients and 5 healthy controls	Japanese	Astrocyte cells (hAST-AQP4) exposure to human sera/Qrt-PCR, Western blot and Immunocytochemistry	NMO sera had a cytotoxic and harmful effect on astrocyte cells. Also decreased d AQP4 mRNA and protein levels while increased IL-6 and TNF-a in astrocytes.	(132)
AQP4IL-6	10 NMOSD patients and 10 healthy controls	Chinese	Astrocyte cells exposed to human sera/Western blot, qRT-PCR, and ELISA	NMO sera downregulated AQP4 levels on the astrocyte surfuce and induced JAK1/2/STAT3-dependent inflammatory response through IL-6 expression.	(133)
Immune responsiveness to *Escherichia coli* (EC), *Staphylococcus aureus* (SA) and *Candida albicans* (CA)	20 NMO patients and 20 healthy controls	Brazilian	PBMC exposed to EC, SA, and CA/flowcytometry and ELISA	Upregulation of IL-1b, IL-6, IL-17, and CD4+ T-cell proliferation, which correlated with neurological disability and downregulation of IL-10 represented in NMO-derived EC-stimulated cell cultures. Increase in LPS levels was reported in plasma of NMO patients.	(134)
MMP-2MMP-9claudin-5VCAM-1	14 NMOSD patients and 10 healthy controls	Japanese	BMECs, astrocytes, and FH-BNBs cells treated with human sera in presence of MMPs inhibitor/ELISA	MMP-2/9 and VCAM-1 secretion was increased in BMECs after exposure to NMOSD sera that led to increased BBB permability.	(125)
AQP4GFAPmyelin immunoreactivity	AQP4+ NMOSD patients	__	Spinal cord slice cultures of null AQP4 mice treated with NMOSD SCF and serum	AQP4-IgG bound to astocytes in spinal cord slice cultures and led to a decrease in AQP4, GFAP, and myelin. NMO lesion was more severe according to increase in specific immune cells and cytokines.	(135)
Eosinophil	NMO patients	__	Eosinophils cultured from mouse bone marrow exposed to NMO sera	Eosinophils induced antibody-dependent cell-mediated cytotoxicity in AQP4-expressed cells and through complement-dependent cell-mediated cytotoxicity led to killing cells.	(136)
27 cytokines/chemokines	20 NMOSD patients and 10 healthy controls	Japanese	BMECs treated with human sera/multiplexed fluorescent bead-based immunoassay system and ELISA	IL-6, MCP-1, and IP-10 were significantly upregulated in BMECs treated with NMOSD acute phase sera. IP-10 levels were correlated with CSF/serum albumin ratio.	(137)
T-cell functions	20 NMO patients and 20 healthy controls	Brazilians	PBMC, CD4-free PBMC, and purified CD4+ T cells cultured and exposed to glucocorticoid inhibitor/flow cytometry and ELISA	T-cell proliferation and Th1 cytokine production were significantly lower in NMO cell cultured, while Th17-like phenotype, IL-6, and IL-23 production were increased. IL-6, IL-21, and IL-23 secretion were less sensitive to glucocorticoid inhibitor.	(138)

## Discussion

NMO comprises a group of immune-meditaed conditions with complex etiology. While family studies have shown clustering of NMO cases in some familites, the exact genetic background of this disorder has not been clarified yet. Since the first report of familial NMO cases in 1936 (14), several studies have attempted to find susceptibility loci for NMO. The first attempts have been focused on the HLA region, based on the importance of this region in the regulation of immune responses and their association with MS, a disorder that clinically resembles NMO. However, various studies have shown that HLA-related susceptibility loci for NMO is distinct from MS. The HLA-DRB1*03 allele has been the mostly appreciated risk locus for NMO. Several other HLA-DRB1, DQB1, and DPB1 alleles have been found to be associated with NMO. Yet, the results of these studies have not been validated in independent cohorts from different ethnic backgrounds.

Exome sequencing and genome-wide SNP arrays have also validated the significance of the HLA region in conferring risk of NMO. In addition, they have shown other risk loci within *AQP4*, *CYP27B1*, *CYP7A1*, *CD226*, *CD58*, *CD6*, *FCRL3*, *GPC5*, *MIF*, *ATG5*, *PD-1.3*, *IL2RA*, *IL7RA*, and *IL17A*. With the exception of *AQP4* and *CD58*, almost other genes have been assessed in single studies, needing confirmation in independent cohorts. Moreover, a number of variants, particularly within *SLC28A3* and *SLC29A1*, have been associated with clinical course or some immune markers in patients with NMO.

Deletion-type CNVs can also been regarded as predisposing factors for NMO. Notably, these CNVs have been found to occur as somatic changes.

In addition to several cytokines that are altered in the course of NMO development, expressions of numerous mRNAs, lncRNAs, and miRNAs have been found to be deregulated in the peripheral blood or brain lesions of NMO patients. Not surprisingly, these genes are mostly enriched in pathways related to functions of the immune system.

Finally, *in vitro* studies have shown the effects of NMO sera on deregulation of function of astrocytes, suggesting the impact of humoral responses on pathoetiology of this condition. Moreover, these circulatory markers could negatively affect permeability of the blood–brain barrier.

Taken together, NMO has a complex genetic background with prominent roles of immune-related genes, particularly cytokine coding genes and those coding cytokine receptors. Future genome-wide studies in NMO patients from different ethnic background would facilitate identification of risk loci for this condition. Finally, systematic review and meta-analysis studies are recommended to produce quantitative results without any bias along with an overview of genetic aspects of disease. Also, further studies should assess treatment responses in association with distinct genetic backgrounds. Finally, a limitation of studies conducted in this filed is that the expression profiles of genes and cytokines have not been assessed in association with different treatment options.

## Author Contributions

MT and SG-F wrote the draft and revised it. TA collected the tables and data. All authors contributed to the article and approved the submitted version.

## Conflict of Interest

The authors declare that the research was conducted in the absence of any commercial or financial relationships that could be construed as a potential conflict of interest.

## Publisher’s Note

All claims expressed in this article are solely those of the authors and do not necessarily represent those of their affiliated organizations, or those of the publisher, the editors and the reviewers. Any product that may be evaluated in this article, or claim that may be made by its manufacturer, is not guaranteed or endorsed by the publisher.
